# Development of an RT-LAMP-CRISPR/Cas12a-based nucleic acid detection platform for rapid diagnosis of porcine reproductive and respiratory syndrome virus type 2

**DOI:** 10.1016/j.vas.2026.100836

**Published:** 2026-08-22

**Authors:** Congcong Li, Changsheng Sun, Qiuliang Xu

**Affiliations:** a College of Animal Science and Technology, Henan University of Animal Husbandry and Economy, Zhengzhou, Henan, China; b Henan Pig Bio-breeding Research Institute, Zhengzhou, Henan, China; c Henan Livestock and Poultry Genetic Resources Protection Engineering Technology Research Center, Zhengzhou, China; d Henan Key Laboratory of Healthy Breeding and Efficient Reproduction of Livestock and Poultry, Zhengzhou, China

**Keywords:** PRRSV-2, RT-LAMP, CRISPR/Cas12a, Nucleic acid detection, Visualization

## Abstract

Porcine reproductive and respiratory syndrome virus (PRRSV) significantly affects swine production, leading to reproductive failure and respiratory disorders. Current detection methods are limited by slow turnaround times, insufficient sensitivity and specificity, the need for skilled personnel, and costly equipment, which hinder their application in rapid on-site diagnosis and early disease prevention and control. In this study, a novel detection platform was developed by integrating the target specificity of RT-LAMP primers with the dual recognition capability of CRISPR/Cas12a. Optimal crRNA primers targeting conserved sequences in the PRRSV-2 structural protein M gene were designed and screened. Reaction conditions were subsequently optimized, and a cloning vector containing the target gene was constructed. Sensitivity evaluation using PCR, LAMP-CRISPR/Cas12a, and qPCR revealed detection limits of 9.17 × 10^3^ copies/μL, 9.17 × 10^-1^ copies/μL, and 9.17 × 10^2^ copies/μL for the target plasmid DNA, respectively. Notably, the LAMP-CRISPR/Cas12a assay exhibited the highest sensitivity, with a detection capability 10⁴-fold and 10³-fold greater than that of PCR-CRISPR/Cas12a and qPCR, respectively. Specificity analysis demonstrated that only HP-PRRSV generated a positive signal among the four tested porcine viruses, including PEDV G2b, PRV HNJY, and PPV SD-1. No fluorescent signals were detected for the other viral strains, indicating the absence of cross-reactivity. Fifty porcine serum samples were analyzed to compare the diagnostic performance of the RT-LAMP-CRISPR/Cas12a assay with that of qPCR, and the results showed complete agreement between the two methods. The established RT-LAMP-CRISPR/Cas12a detection system provides a rapid and reliable approach for PRRSV detection and may facilitate improved surveillance and control of PRRSV infections.

## Highlights

• RT-LAMP-CRISPR/Cas12a assay rapidly detects PRRSV-2 M gene.
• LoD of 9.17 × 10⁻¹ copies/μL for target plasmid DNA.
• Specific to PRRSV; no cross-reaction with other swine viruses.
• 100% concordance with qPCR in 50 clinical samples (70% positive).

## 1 Introduction

Porcine reproductive and respiratory syndrome (PRRS) is an immunosuppressive disease caused by porcine reproductive and respiratory syndrome virus (PRRSV), affecting pigs of all ages and characterized by reproductive disorders and respiratory symptoms (Raymond et al., 2017). Since its first identification in the United States in 1987, PRRSV has emerged as one of the most economically devastating pathogens affecting the global swine industry. Following the first report of PRRS in China in 1995, porcine reproductive and respiratory syndrome virus type 2 (PRRSV-2; North American type) has become the predominant genotype circulating in Chinese swine populations. A large-scale molecular epidemiological survey conducted in Sichuan Province from 2021 to 2023 revealed that PRRSV-2 accounted for 95.65% (615/643) of PRRSV-positive samples, whereas PRRSV-1 represented only 4.35% (28/643) (Huang et al., 2024). Although PRRSV-1 has been detected in at least 28 regions in China (Qiu et al., 2025), its prevalence remains substantially lower than that of PRRSV-2. The rapid expansion of the Chinese pig industry and the widespread implementation of high-density farming systems have facilitated PRRSV transmission and evolution. The persistent circulation of PRRSV in China, together with increasing genetic diversity and antigenic variation, continues to pose significant challenges for effective disease prevention and control. PRRSV belongs to the family Arteriviridae within the order Nidovirales (Chaudhari and Vu, 2020). Pigs are the only natural hosts of PRRSV, which primarily targets alveolar macrophages (Wensvoort et al., 1991). PRRSV is an enveloped, single-stranded positive-sense RNA virus with an approximately 15 kb genome encoding eight structural proteins and at least 16 non-structural proteins (Khatun et al., 2019; Zhou et al., 2015). Its high genetic diversity, prolonged persistence, and multiple transmission routes, including airborne, direct contact, and vertical transmission, contribute to delayed immune responses and sustained viremia, thereby promoting viral dissemination. Moreover, the lack of rapid and convenient diagnostic methods, combined with overlapping clinical manifestations with other swine diseases such as brucellosis, pseudorabies, and porcine parvovirus (PPV) infection, makes accurate PRRSV identification challenging. Several genomic regions of PRRSV have been investigated as targets for nucleic acid detection. Open reading frames (ORFs) 2–5 encode major glycoproteins (GP2a, E, GP3, GP4, GP5, and GP5a), ORF6 encodes the matrix protein M, and ORF7 encodes the nucleocapsid protein N. Among these targets, the M gene represents one of the most conserved regions within the PRRSV genome (Kapur et al., 1996), with M protein amino acid sequence homology reaching 96–100% among diverse virulent North American PRRSV isolates (Meng et al., 1995). Therefore, the M gene was selected as the target sequence for developing rapid, accurate, and efficient detection techniques to facilitate timely diagnosis and control of PRRSV infection. Conventional diagnostic methods, including virus isolation, antigen detection, and molecular assays, are often limited by lengthy procedures, insufficient sensitivity and specificity, the requirement for specialized personnel, and dependence on expensive equipment. These limitations restrict their application in rapid point-of-care diagnosis and early PRRSV prevention and control.

Molecular biology-based detection methods, including conventional reverse transcription polymerase chain reaction (RT-PCR) (Li et al., 2017; Yang et al., 2013), real-time quantitative PCR (qPCR) (Chai et al., 2013; Zhang et al., 2022; Zheng et al., 2020), digital PCR (dPCR) (Yang et al., 2017), isothermal amplification techniques such as loop-mediated isothermal amplification (LAMP) and recombinase polymerase amplification (RPA) (Gou et al., 2014; Park et al., 2016; Wang et al., 2017; Yang et al., 2016), metagenomic next-generation sequencing (mNGS), and emerging CRISPR-based technologies represent the current mainstream approaches for PRRSV detection. Conventional PCR provides high sensitivity and specificity but is labor-intensive and may generate false-negative results due to viral genetic variation. qPCR improves detection sensitivity and enables viral load quantification; however, it requires expensive precision instruments and specific primers that may be affected by viral mutations. dPCR exhibits superior sensitivity for detecting low-abundance viral nucleic acids but relies on costly and complex equipment, limiting its widespread application (Hindson et al., 2011; Yang et al., 2017). Isothermal amplification technologies, including LAMP and RPA, enable rapid and convenient on-site detection with minimal equipment requirements, but their relatively lower specificity increases the risk of nonspecific amplification and false-positive results (Wernike et al., 2012). Metagenomic high-throughput sequencing enables the identification of unknown viruses and emerging variants while providing comprehensive genomic information (Chen et al., 2020; Chen et al., 2022; Tan et al., 2019). However, its high cost, complex bioinformatic analysis, and prolonged turnaround time restrict its application primarily to research settings. The clustered regularly interspaced short palindromic repeats (CRISPR)/CRISPR-associated (Cas) system, which evolved as an adaptive immune defense mechanism in bacteria and archaea, has facilitated the development of nucleic acid detection platforms based on collateral cleavage activity (Paul and Montoya, 2020). CRISPR-associated protein 12a (Cas12a) enables DNA detection through collateral cleavage of single-stranded DNA reporters, termed DNA endonuclease-targeted CRISPR trans reporter (DETECTR) (Chen et al., 2018), whereas CRISPR-associated protein 13a (Cas13a) detects RNA targets using fluorescently labeled RNA reporters, termed Specific High-sensitivity Enzymatic Reporter UnLOCKing (SHERLOCK) (Kellner et al., 2019). However, both approaches generally require pre-amplification by isothermal amplification methods to improve sensitivity for low-abundance nucleic acid targets. The integration of CRISPR/Cas systems with isothermal amplification technologies has resulted in portable detection platforms with enhanced specificity, sensitivity, and operational convenience. Furthermore, researchers have improved the specificity of PRRSV detection by combining CRISPR-based methods with RPA amplification (Chang et al., 2020; Liu et al., 2021).

Currently, PRRSV detection mainly relies on qPCR, which is limited by relatively high detection limits (10¹–10² copies/μL), lengthy processing times (3-4 h), and the requirement for expensive equipment. In this study, a novel PRRSV-2 detection method was established by integrating RT-LAMP with CRISPR/Cas12a. This assay exploits the synergistic advantages of LAMP-mediated nucleic acid amplification and Cas12a-based precise target recognition, achieving a lower detection limit (9.17 × 10⁻¹ copies/μL), a shorter detection time (80-150 min), and simplified operation without the need for complex instrumentation. Moreover, the detection results can be directly visualized by the naked eye under blue light, ultraviolet (UV) light, or even natural light. The objectives of this study were to establish the RT-LAMP-CRISPR/Cas12a assay, optimize key reaction parameters (including temperature, incubation time, deoxyribonucleotide triphosphate (dNTP) concentration, and Mg²⁺ concentration), evaluate analytical sensitivity and specificity, and validate the assay using 50 clinical serum samples with qPCR as the reference method. This integrated platform reduces the risk of nonspecific detection and improves the sensitivity and accuracy of target nucleic acid identification. It provides an innovative approach for rapid and reliable PRRSV-2 diagnosis, particularly for on-site screening in resource-limited settings, such as primary veterinary clinics and large-scale pig farms. Additionally, it may serve as a complementary diagnostic tool in veterinary diagnostic laboratories.

## 2 Materials and methods

### 2.1 Virus and samples

The nucleic acid isolates of pseudorabies virus (PRV) HNJY, PPV SD-1, highly pathogenic porcine reproductive and respiratory syndrome virus (HP-PRRSV) Lineage 8, and porcine epidemic diarrhea virus (PEDV) G2b used in this study were obtained from the laboratory strain repository. A total of 50 serum samples were collected from pigs of the same batch at 56 days of age exhibiting respiratory symptoms, including fever, coughing, and wheezing, at a commercial pig farm in Henan Province. Viral RNA/DNA was extracted from the samples using a commercial virus RNA/DNA extraction kit, and the extracted nucleic acids were stored under appropriate conditions for subsequent molecular analyses.

### 2.2 Primer design

PCR primers, LAMP primers, and single-guide RNA (crRNA) sequences were designed based on the conserved region of the PRRSV-2 structural protein M gene (GenBank accession number: NC_001961.1). The crRNA, PCR primer, and LAMP primer designs were performed using CRISPR-offinder software (Zhao et al., 2017), Primer5 (Premier Biosoft), and LAMP Designer 1.16 (Premier Biosoft), respectively. All primers were synthesized and purified by Shanghai Shenggong Bio-technology Co., Ltd. Detailed primer sequences, genomic positions, and information on the PCR and LAMP amplification products are provided in Supplementary Tables 1 and 2.

### 2.3 Extraction of viral RNA and construction of plasmids

Viral RNA was extracted from virus-infected cells using a viral RNA extraction kit (B518667, Shanghai Shenggong Bio-technology Co., Ltd.) according to the manufacturer's instructions. Complementary DNA (cDNA) was synthesized from the extracted RNA using a reverse transcription kit (K1622, Thermo Fisher Scientific). The M gene fragment was subsequently amplified by PCR using cDNA as the template. The amplified PCR product was purified, ligated into the pMD-19T vector, and transformed into competent *Escherichia coli* cells. Positive clones were screened by monoclonal colony PCR and confirmed by Sanger sequencing. Confirmed recombinant clones were cultured, and the recombinant plasmid pMD19T-M was extracted for subsequent experiments.

### 2.4 *In vitro* transcription and screening of crRNA

A forward primer containing the T7 promoter and specific reverse primers of different lengths targeting distinct regions were used to amplify crRNA templates from the pUC57-T7-crRNA plasmid by PCR. The amplified PCR products were purified using a PCR purification kit and subsequently used as templates for *in vitro* transcription with T7 RNA polymerase. The transcribed crRNAs were purified using an RNA purification kit, and their concentration and purity were determined by spectrophotometry. Seven crRNAs targeting different regions of the PRRSV structural protein M gene were designed and synthesized. The crRNAs were diluted, and the Cas12a cleavage reaction system was prepared as follows: 2 μL of purified PCR product, 2 μL of NEB buffer 2.1, 250nM of Cas12a enzyme, 132 ng of crRNA, 2.5 μM 6-carboxy-4′,5′-dichloro-2′,7′-dimethoxyfluorescein (JOE) probe, with the final volume adjusted to 20 μL using nuclease-free water. The reaction mixture was incubated in a real-time quantitative PCR (qPCR)instrument at 37°C for 30 min. The optimal crRNA was selected based on the highest fluorescence intensity detected under different light sources.

JOE (Ex/Em = 515/545 nm) and ROX (Ex/Em = 565/605 nm) were selected as fluorescent reporters. Their emission spectra are non‑overlapping, allowing independent green and red fluorescence signals, with visible color changes to the naked eye upon cleavage. JOE is compatible with portable blue light transilluminators, and ROX is discernible under natural light, collectively covering multiple visualization conditions for resource‑limited settings.

### 2.5 Development of a detection system combining LAMP with CRISPR/Cas12a

To optimize the cleavage efficiency of the LAMP-CRISPR/Cas12a system, single-factor optimization experiments were performed by evaluating different reaction temperatures (61–69°C), incubation times (15–55 min), dNTP concentrations (1–1.8 mM), and Mg²⁺ concentrations (2–10 mM). For JOE and ROX fluorescent probes, cleavage times ranging from 5 to 30 min were assessed. Each experimental condition was tested in triplicate. The optimal reaction parameters were determined based on fluorescence signal intensity. The LAMP reaction mixture consisted of 12.5 μL of WarmStart LAMP 2 × Master Mix (NEB, USA), or alternatively 8 U of 10 × Bst DNA polymerase, 2.5 μL of 10 × Bst buffer, 4 mM MgSO₄, and 1.2 mM dNTPs. The final concentrations of LAMP primers were 1.6 μM for forward inner primer/backward inner primer (FIP/BIP), 0.4 μM for forward outer primer/backward outer primer (F3/B3), and 0.2 μM for loop forward primer/loop backward primer (LF/LB). A total of 2.0 μL of plasmid template DNA was added, and the final volume was adjusted to 25 μL with nuclease-free water. The LAMP reaction was performed at 64°C for 50 min. For CRISPR/Cas12a detection, 2 μL of LAMP products was added to a reaction mixture containing 250nM of Cas12a enzyme, 2 μL of NEB buffer 2.1, 2.5 μM JOE probe or 15 μM 6-carboxy-X-rhodamine (ROX) probe, and 132 ng of crRNA. The final volume was adjusted to 20 μL with nuclease-free water, followed by incubation at 37°C for 30 min. Fluorescence signals were subsequently evaluated using blue light illumination, UV light visualization with a gel imaging system, and a qPCR instrument.

### 2.6 Sensitivity analysis

For the qPCR assay, the same primers used for conventional PCR were applied (Supplementary Table 1). The assay was validated by standard curve analysis, showing an amplification efficiency of 102.067%, an R² value of 0.996, and a slope of −3.273. The standard curve exhibited a linear dynamic range from 9.17 × 10⁸ to 9.17 × 10² copies/μL. The qPCR reaction mixture (15 μL) contained 7.5 μL of 2 × SYBR Green Master Mix, 0.5 μL of each primer, and 5 μL of plasmid DNA template. Amplification was performed on a QuantStudio 6 Pro system with an initial denaturation step at 95°C for 10 min, followed by 40 cycles of denaturation at 95°C for 15 s and annealing/extension at 60°C for 60 s. Melting curve analysis was performed from 60°C to 95°C for all reactions to confirm amplification specificity. A no-template control (NTC) containing nuclease-free water was included in each run. The cycle threshold (CT) was used to determine the presence of target nucleic acid.

The plasmid copy number was calculated using the following formula:

Copies/μL = (6.023 × 10²³ × concentration (ng/μL) × 10⁻⁹) / [plasmid length (bp) × 660].

The total plasmid length, including the inserted target fragment and vector backbone, was 3,142 bp. Three independent serial dilutions were prepared and analyzed for each concentration level.

### 2.7 Specific detection

The specificity of the LAMP-CRISPR/Cas12a detection system was evaluated using four porcine viruses: PRV, PPV, PRRSV, and PEDV. Viral nucleic acids were extracted and subjected to LAMP or reverse transcription LAMP (RT-LAMP) amplification, followed by target detection using the Cas12a-mediated cleavage system. Nuclease-free water was included as a negative control to monitor potential contamination and nonspecific signals.

### 2.8 Detection of serum samples

Viral RNA was extracted from collected serum samples using a viral RNA extraction kit (Takara, Japan) and reverse transcribed into complementary DNA (cDNA). The obtained cDNA was diluted 3-fold and used as the template for detection using the LAMP-CRISPR/Cas12a system. The recombinant plasmid pMD19T-M was used as a positive control. In addition, serum sample-derived cDNA was analyzed by qPCR to evaluate the consistency and performance of the LAMP-CRISPR/Cas12a assay.

### 2.9 Statistical analysis

All experiments were independently performed in triplicate, and the data were presented as mean ± standard deviation (SD). The assumptions for one-way analysis of variance (ANOVA) were evaluated using the Shapiro–Wilk test for normality and the Levene test for homogeneity of variances. In sensitivity analyses, differences in fluorescence intensity among plasmid concentration gradients were assessed using one-way ANOVA. A four-parameter logistic (4PL) nonlinear regression model was applied to fit the dose–response relationship between the logarithmically transformed plasmid concentration (log₁₀ copies/μL) and fluorescence intensity (relative fluorescence units, RFU). The "Log(agonist) vs. response" module in GraphPad Prism 9.0 was used for least-squares fitting, calculation of the half-maximal effective concentration (EC₅₀) with its 95% confidence interval (CI), and estimation of the goodness-of-fit parameter (R²). The limit of detection (LoD) was defined according to the Clinical and Laboratory Standards Institute (CLSI) EP12 guideline as the lowest concentration at which all tested replicates produced positive results, independent of 4PL model extrapolation. For specificity analysis, differences in fluorescence intensity among different viral groups were evaluated using one-way ANOVA. When significant differences were detected, Dunnett’s multiple comparison test was performed to compare each virus group with the negative control group while controlling the family-wise error rate at α = 0.05.

All statistical analyses were performed using GraphPad Prism 9.0 (GraphPad Software, San Diego, CA, USA) and IBM SPSS Statistics 27.0 (IBM Corp., Armonk, NY, USA). A two-sided P value < 0.05 was considered statistically significant.

For clinical sample validation, the detection results obtained by the RT-LAMP-CRISPR/Cas12a assay and qPCR were summarized using a 2 × 2 contingency table (confusion matrix). The 95% CIs for positive percent agreement (PPA), negative percent agreement (NPA), and overall percent agreement (OPA) were calculated using the exact Clopper-Pearson method.

## 3 Results

### 3.1 Identification of the optimal crRNA targeting the PRRSV M gene for the CRISPR/Cas12a system

**Fig. 1:**
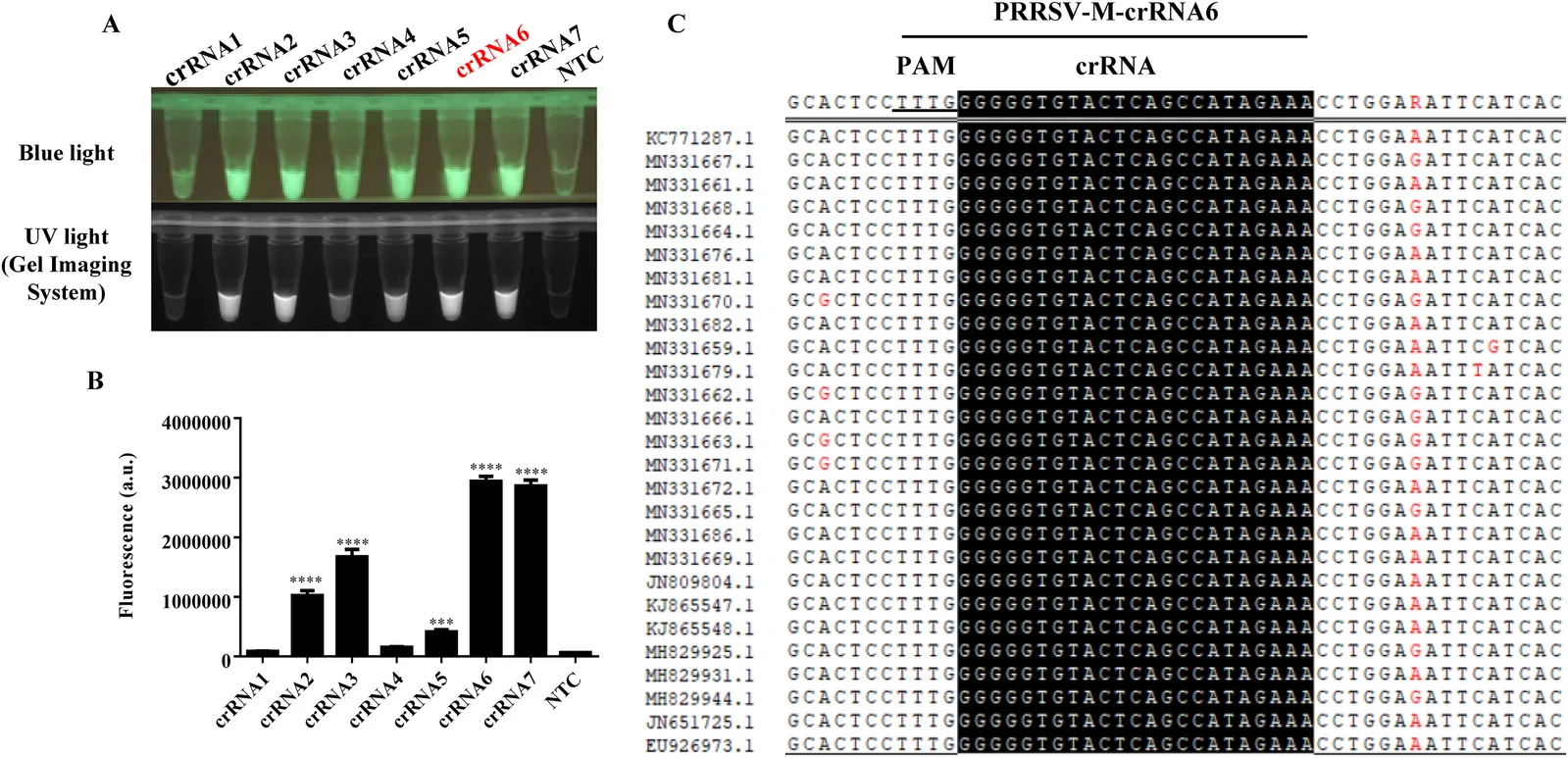
Selection of the optimal crRNA. (A) Cas12a-mediated cleavage of PRRSV crRNAs under different excitation wavelengths. (B) Histogram showing fluorescence signals generated by Cas12a-mediated cleavage of different PRRSV crRNAs (1–7). (C) Conservation analysis of PRRSV-M-crRNA6 among 27 PRRSV-2 strains. Error bars represent the SD of fluorescence intensity obtained from three independent reactions. NTC, no-template control; SD, standard deviation; crRNA, CRISPR RNA; PRRSV, porcine reproductive and respiratory syndrome virus. ****P<0.0001, *** *P*<0.001.

The Cas12a-mediated cleavage reactions were monitored in real time using a qPCR instrument. Fluorescence signals were evaluated and compared under blue light, UV light, and gel imaging system illumination. Among the seven designed crRNAs, crRNA6 produced the strongest fluorescence signal, exhibiting the highest fluorescence intensity and brightness (Fig. 1A, B). Sequence alignment analysis of 27 reference PRRSV-2 strains representing different lineages demonstrated that the target region recognized by crRNA6 was 100% conserved among all analyzed sequences (Fig. 1C). These results suggest that crRNA6 has broad recognition potential for PRRSV-2 strains and was therefore selected for subsequent experiments.

### 3.2 Optimization of reaction conditions for the LAMP-CRISPR/Cas12a system

**Fig. 2:**
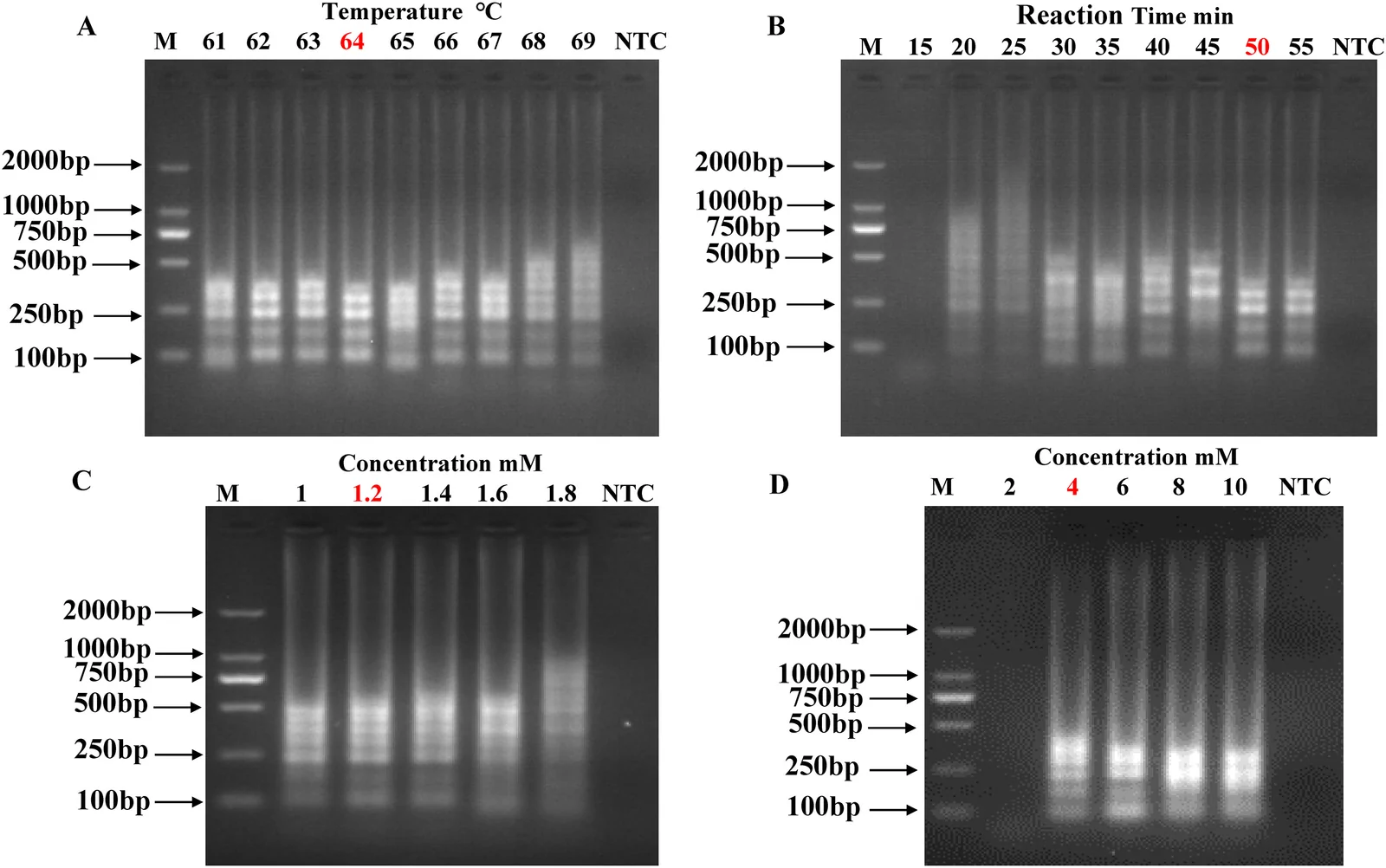
Optimization of LAMP reaction conditions. (A) Optimization of the incubation temperature for the LAMP reaction. (B) Optimization of the LAMP amplification time. (C) Optimization of dNTP concentration. (D) Optimization of Mg²⁺ concentration. NTC, no-template control; M, DL 2000 DNA Marker; LAMP, loop-mediated isothermal amplification; dNTP, deoxyribonucleotide triphosphate.

**Fig. 3:**
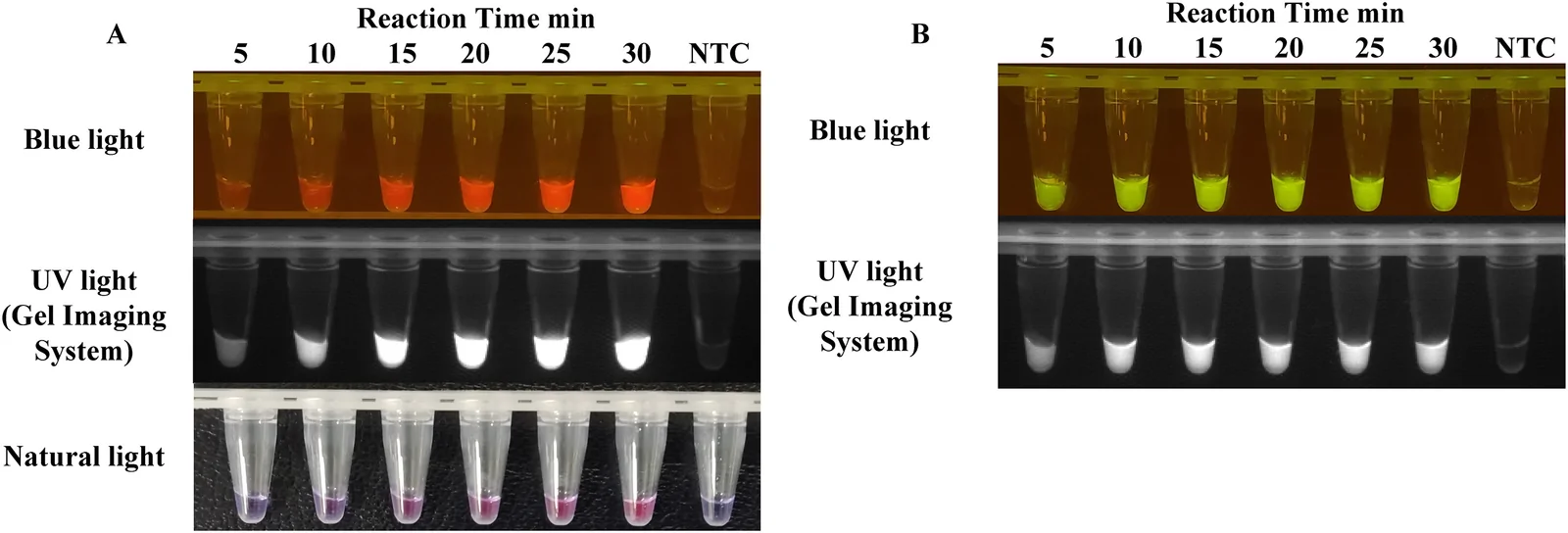
Optimization of Cas12a-mediated cleavage time. (A) Detection results of the ROX probe at different Cas12a cleavage times. (B) Detection results of the JOE probe at different Cas12a cleavage times. NTC, no-template control; ROX, 6-carboxy-X-rhodamine; JOE, 6-carboxy-4′,5′-dichloro-2′,7′-dimethoxyfluorescein.

The recombinant plasmid pMD19T-M was used as the template for optimization of the LAMP reaction conditions. To determine the optimal amplification temperature, LAMP reactions were performed at temperatures ranging from 61°C to 69°C. Gel electrophoresis analysis showed that the most intense and specific amplification bands were obtained at 64°C (Fig. 2A). To optimize the amplification duration while minimizing nonspecific amplification, reaction times ranging from 15 to 55 min were evaluated. The strongest amplification products were observed after 50 min of incubation (Fig. 2B). Subsequently, the concentrations of dNTPs and Mg^2+^ were individually optimized to achieve optimal amplification efficiency, sensitivity, and specificity. The results indicated that 1.2 mM dNTPs and 4 mM Mg²⁺ represented the optimal concentrations for the LAMP reaction (Fig. 2C, D). To determine the optimal cleavage time for the CRISPR/Cas12a detection step, incubation periods ranging from 5 to 30 min were evaluated. Both ROX and JOE fluorescent probes generated detectable fluorescence signals after 10 min of Cas12a-mediated cleavage (Fig. 3A, B).

### 3.3 Evaluation of the sensitivity of LAMP-CRISPR/Cas12a for detection of the PRRSV M gene

**Fig. 4:**
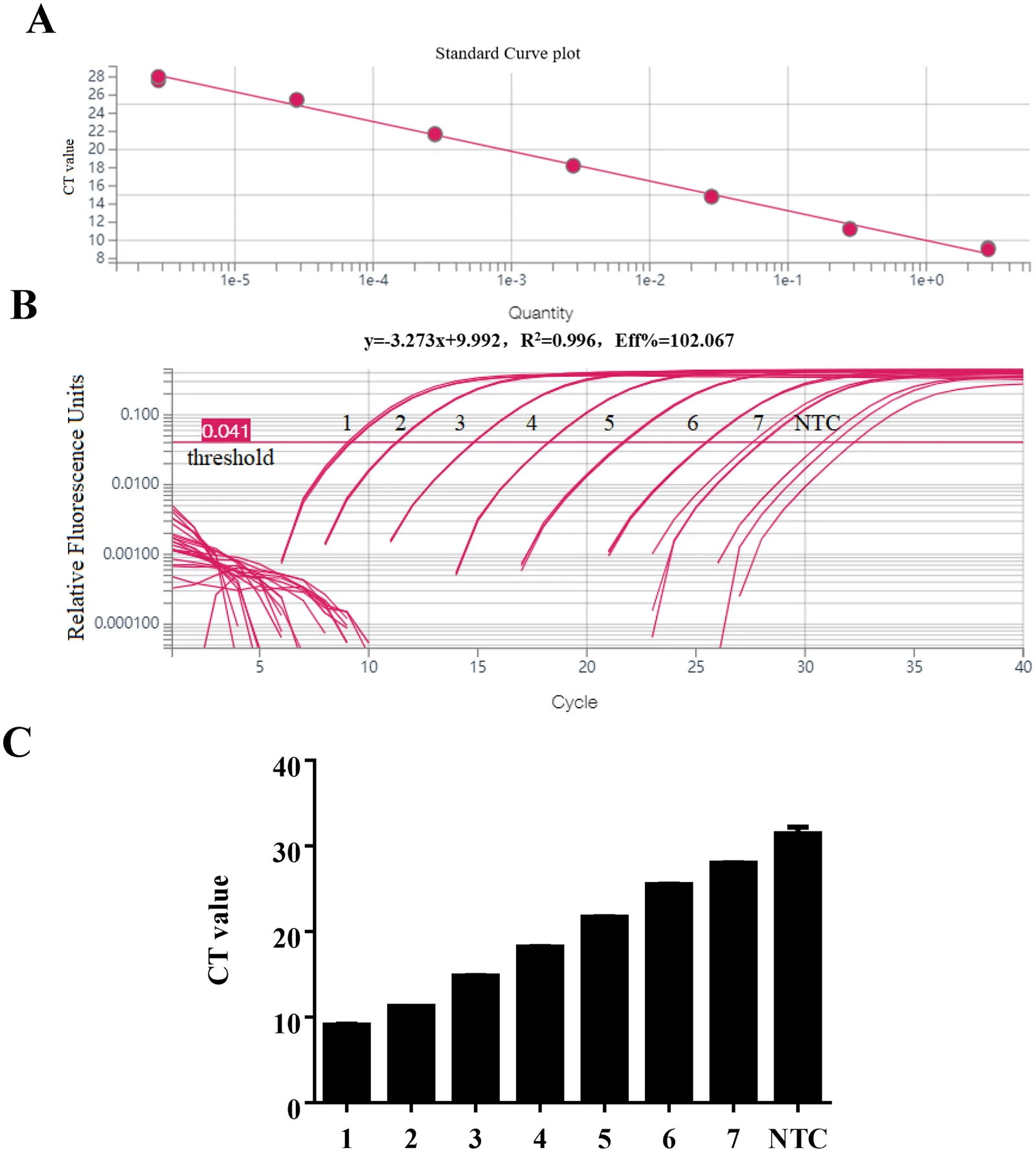
Sensitivity analysis of qPCR detection. (A) Standard curve. (B) Amplification curve. (C) CT values obtained from different plasmid concentrations. Plasmid concentrations used as amplification templates (1-7) were 9.17 × 10^8^ copies/μL, 9.17 × 10^7^ copies/μL, 9.17 × 10^6^ copies/μL, 9.17 × 10^5^ copies/μL, 9.17 × 10^4^ copies/μL, 9.17 × 10^3^ copies/μL, and 9.17 × 10^2^ copies/μL, respectively. Error bars represent the SD of fluorescence intensity obtained from three independent reactions. NTC, no-template control; qPCR, real-time quantitative polymerase chain reaction; CT, cycle threshold.

**Fig. 5:**
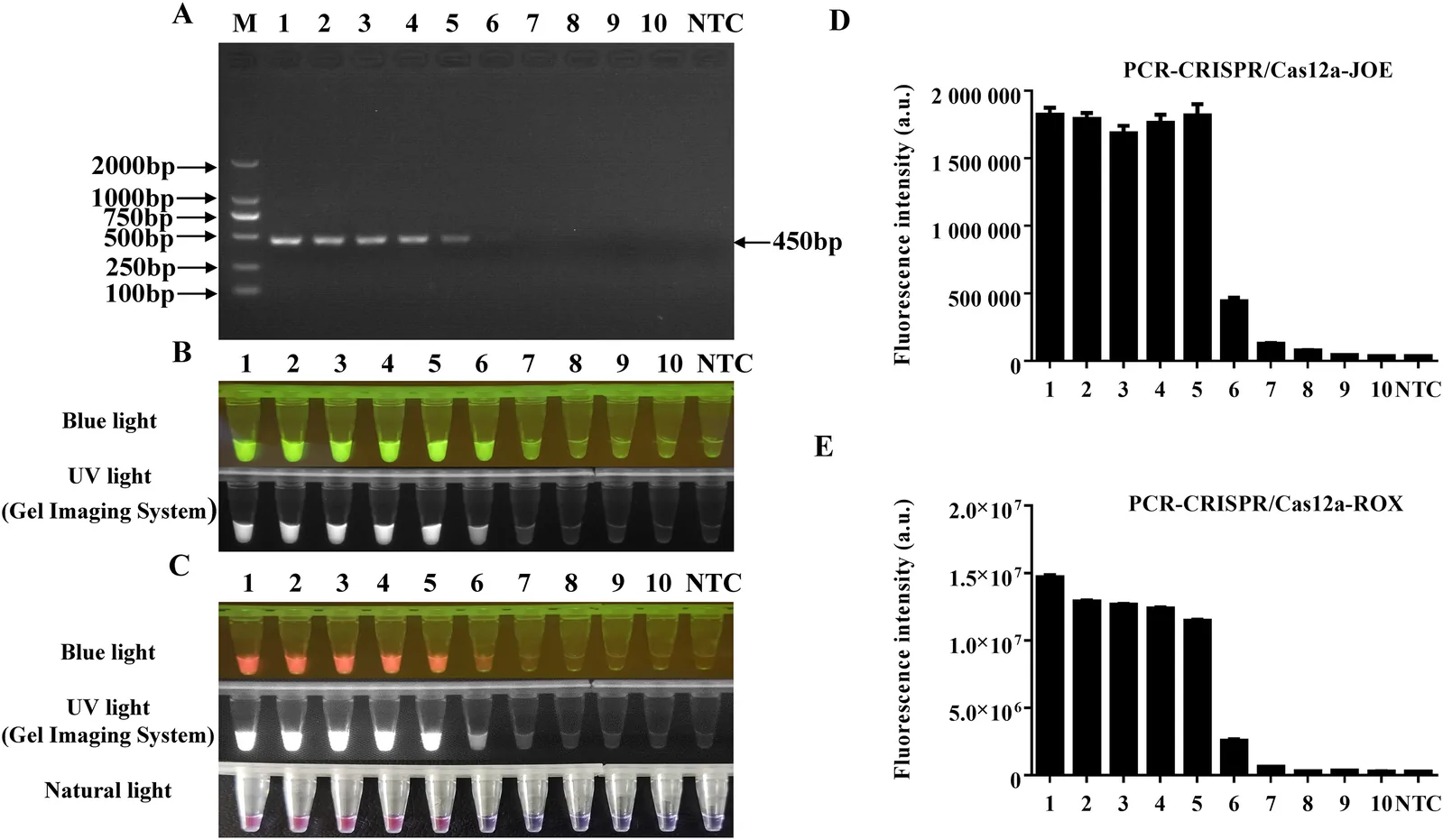
Sensitivity analysis of PCR-CRISPR/Cas12a. (A) Agarose gel electrophoresis analysis of PCR amplification products. (B) Cas12a-mediated cleavage results of PCR-CRISPR/Cas12a-JOE under different excitation wavelengths. (C) Cas12a-mediated cleavage results of PCR-CRISPR/Cas12a-ROX under different excitation wavelengths. (D) Fluorescence intensity of the JOE probe recorded by the qPCR instrument. (E) Fluorescence intensity of the ROX probe recorded by the qPCR instrument. Plasmid concentrations used as amplification templates (1-10) were 9.17 × 10^8^ copies/μL, 9.17 × 10^7^ copies/μL, 9.17 × 10^6^ copies/μL, 9.17 × 10^5^ copies/μL, 9.17 × 10^4^ copies/μL, 9.17 × 10^3^ copies/μL, 9.17 × 10^2^ copies/μL, 9.17 × 10^1^ copies/μL, 9.17 × 10° copies/μL, and 9.17 × 10^-1^ copies/μL, respectively. Error bars represent the SD of fluorescence intensity obtained from three independent reactions. NTC, no-template control; PCR, polymerase chain reaction; CRISPR, clustered regularly interspaced short palindromic repeats; Cas12a, CRISPR-associated protein 12a; JOE, 6-carboxy-4′,5′-dichloro-2′,7′-dimethoxyfluorescein; ROX, 6-carboxy-X-rhodamine; qPCR, real-time quantitative polymerase chain reaction; SD, standard deviation.

**Fig. 6:**
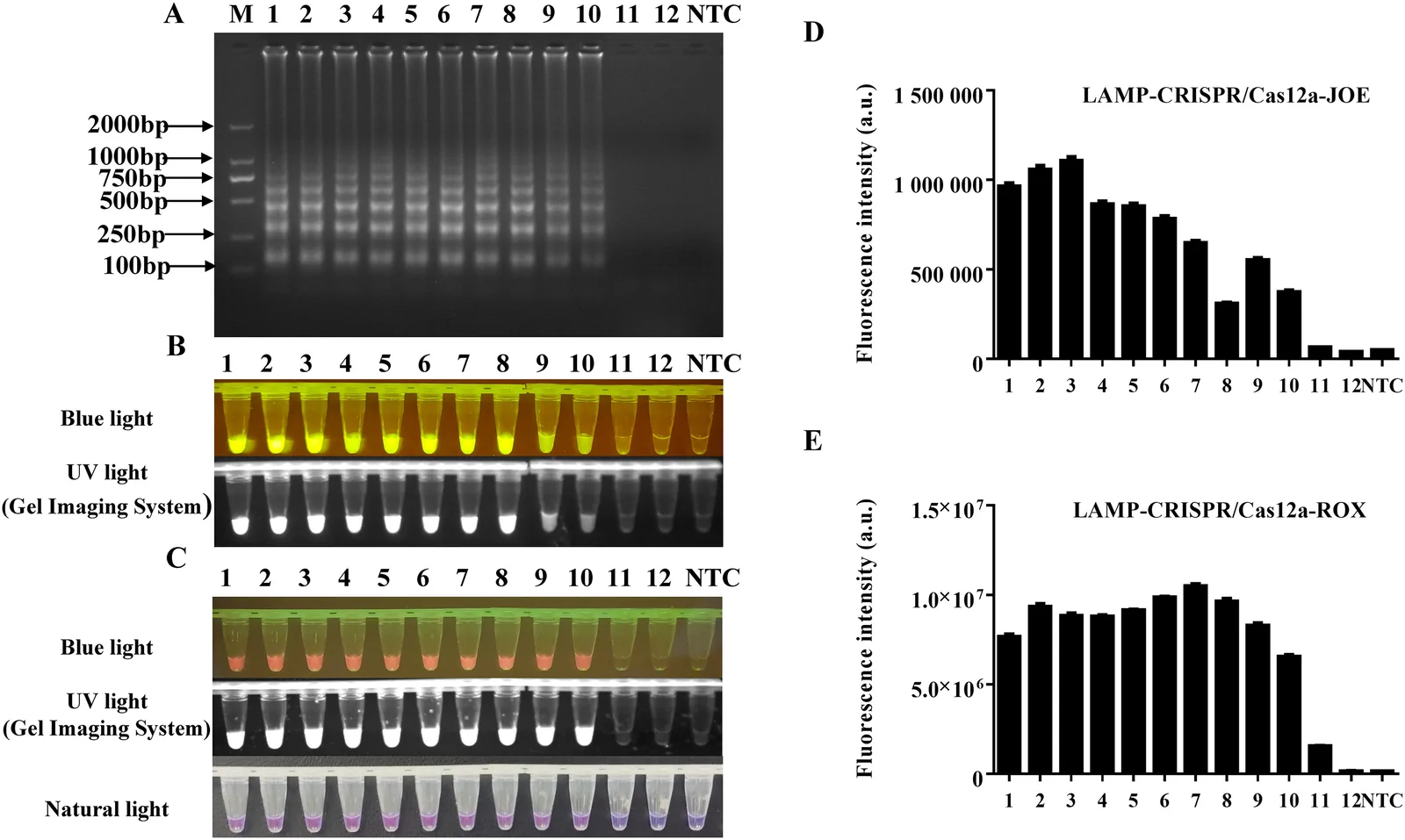
Sensitivity analysis of LAMP-CRISPR/Cas12a. (A) Agarose gel electrophoresis analysis of LAMP amplification products. (B) Cas12a-mediated cleavage results of LAMP-CRISPR/Cas12a-JOE under different excitation wavelengths. (C) Cas12a-mediated cleavage results of LAMP-CRISPR/Cas12a-ROX under different excitation wavelengths. (D) Fluorescence intensity of the JOE probe measured by the qPCR instrument. (E) Fluorescence intensity of the ROX probe measured by the qPCR instrument. Plasmid concentrations used as amplification templates (1-12) were 9.17 × 10^8^ copies/μL, 9.17 × 10^7^ copies/μL, 9.17 × 10^6^ copies/μL, 9.17 × 10^5^ copies/μL, 9.17 × 10^4^ copies/μL, 9.17 × 10^3^ copies/μL, 9.17 × 10^2^ copies/μL, 9.17 × 10^1^ copies/μL, 9.17 × 10° copies/μL, 9.17 × 10^-1^ copies/μL, 9.17 × 10^-2^ copies/μL, and 9.17 × 10^-3^ copies/μL, respectively. Error bars represent the SD of fluorescence intensity obtained from three independent reactions. NTC, no-template control; LAMP, loop-mediated isothermal amplification; CRISPR, clustered regularly interspaced short palindromic repeats; Cas12a, CRISPR-associated protein 12a; JOE, 6-carboxy-4′,5′-dichloro-2′,7′-dimethoxyfluorescein; ROX, 6-carboxy-X-rhodamine; qPCR, real-time quantitative polymerase chain reaction; SD, standard deviation.

The analytical sensitivity of the LAMP-CRISPR/Cas12a assay was evaluated using a 10-fold serial dilution series of the plasmid standard as the template. Three detection approaches were compared: LAMP combined with Cas12a-mediated cleavage, PCR combined with Cas12a-mediated cleavage, and qPCR. The results are presented in Fig. 4, Fig. 5, Fig. 6. The statistical results of one-way ANOVA, Dunnett’s post hoc analysis, and the parameters obtained from four-parameter logistic (4PL) nonlinear regression fitting, including EC₅₀ values and their 95% confidence intervals, are summarized in Supplementary Tables 3 and 4. The detection limits of LAMP-CRISPR/Cas12a, PCR-CRISPR/Cas12a, and qPCR were 9.17 × 10^-1^ copies/μL (Fig. 6, Supplementary Table 5), 9.17 × 10^3^ copies/μL (Fig. 5), and 9.17 × 10^2^ copies/μL (Fig. 4), respectively. Among the evaluated methods, the LAMP-CRISPR/Cas12a assay exhibited the highest analytical sensitivity, with detection capability 10⁴-fold and 10³-fold greater than that of PCR-CRISPR/Cas12a and qPCR, respectively. Furthermore, the LAMP-CRISPR/Cas12a-ROX assay generated strong fluorescence signals with favorable signal-to-noise characteristics, demonstrating its suitability for rapid nucleic acid detection applications.

### 3.4 Evaluation of the specificity of LAMP-CRISPR/Cas12a for detection of the PRRSV M gene

**Fig. 7:**
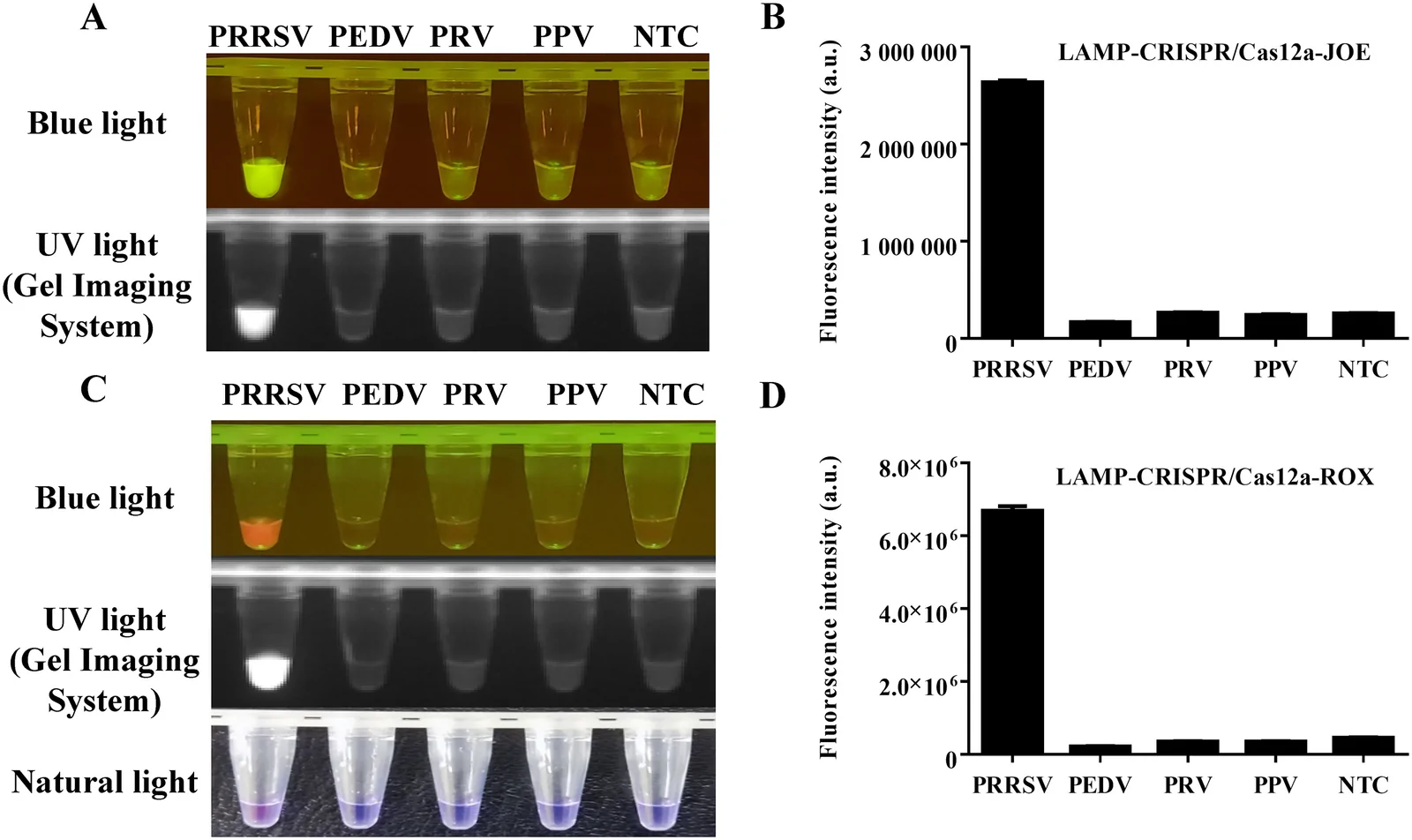
Specificity analysis. (A) Cas12a-mediated cleavage results of LAMP/RT-LAMP-CRISPR/Cas12a-JOE under different excitation wavelengths. (B) Fluorescence intensity values generated by different viruses using LAMP/RT-LAMP-CRISPR/Cas12a-JOE. (C) Cas12a-mediated cleavage results of LAMP/RT-LAMP-CRISPR/Cas12a-ROX under different excitation wavelengths. (D) Fluorescence intensity values generated by different viruses using LAMP/RT-LAMP-CRISPR/Cas12a-ROX. Error bars represent the SD of fluorescence intensity obtained from three independent reactions. The tested viral samples include: HP-PRRSV (lineage 8), PEDV G2b, PRV HNJY, and PPV SD-1, with sterile water as the no-template control (NTC). M, DL 2000 DNA Marker; LAMP, loop-mediated isothermal amplification; RT-LAMP, reverse transcription loop-mediated isothermal amplification; CRISPR, clustered regularly interspaced short palindromic repeats; Cas12a, CRISPR-associated protein 12a; JOE, 6-carboxy-4′,5′-dichloro-2′,7′-dimethoxyfluorescein; ROX, 6-carboxy-X-rhodamine; SD, standard deviation.

The specificity of the LAMP-CRISPR/Cas12a assay was evaluated using four common swine viruses, including HP-PRRSV, PEDV G2b, PRV HNJY, and PPV SD-1. Positive fluorescent signals were detected only in HP-PRRSV samples, whereas no detectable signals were observed for PEDV G2b, PRV HNJY, or PPV SD-1 (Fig. 7, Supplementary Figure 1). These results demonstrated that the assay showed no cross-reactivity among the tested pathogens. The detailed statistical analyses of fluorescence intensity in the ROX and JOE channels, including one-way ANOVA and Dunnett's post hoc comparisons, are provided in Supplementary Table 6.

### 3.5 Application of the LAMP-CRISPR/Cas12a assay in the detection of clinical PRRSV specimens

**Fig. 8:**
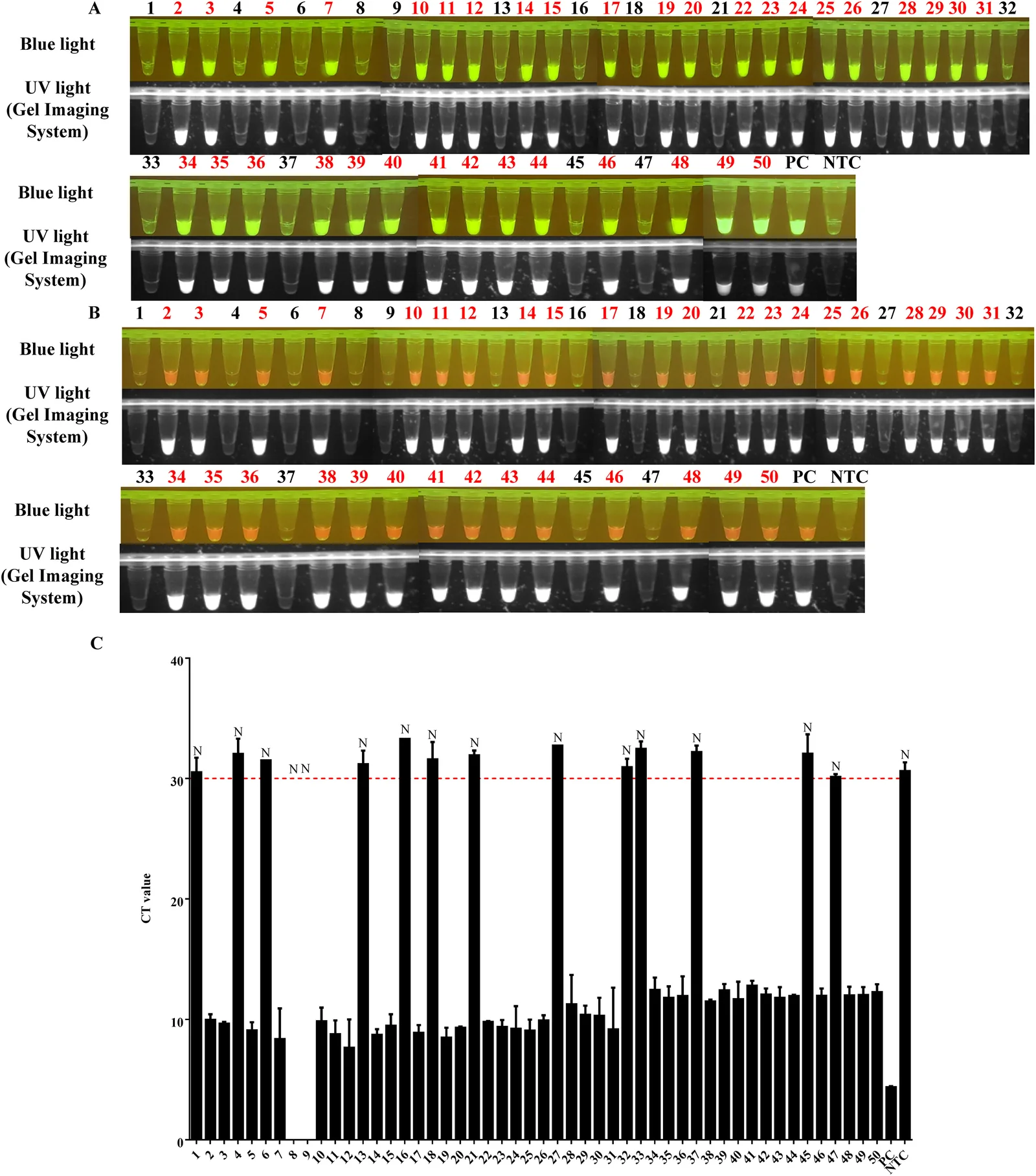
Detection results of porcine serum samples. (A) Cas12a-mediated cleavage results of RT-LAMP-CRISPR/Cas12a-JOE under different excitation wavelengths. (B) Cas12a-mediated cleavage results of RT-LAMP-CRISPR/Cas12a-ROX under different excitation wavelengths. (C) CT values detected by RT-qPCR. Samples 1-50 represent different porcine serum samples. PC and NTC represent positive and no-template controls, respectively. CT values >30 or equal to 0 were considered negative, whereas CT values between 0 and 30 were considered positive. Error bars represent the SD of fluorescence intensity obtained from three independent reactions. NTC, no-template control; PC, positive control; RT-LAMP, reverse transcription loop-mediated isothermal amplification; CRISPR, clustered regularly interspaced short palindromic repeats; Cas12a, CRISPR-associated protein 12a; JOE, 6-carboxy-4′,5′-dichloro-2′,7′-dimethoxyfluorescein; ROX, 6-carboxy-X-rhodamine; qPCR, real-time quantitative polymerase chain reaction; RT-qPCR, reverse transcription quantitative polymerase chain reaction; CT, cycle threshold.

The detection performance of the RT-LAMP-CRISPR/Cas12a assay was evaluated using 50 porcine serum samples, with qPCR used as the reference method. Cas12a-mediated cleavage reactions were performed using JOE (Fig. 8A) and ROX (Fig. 8B) fluorescent probes, and fluorescence intensities were recorded using a qPCR instrument for JOE (Supplementary Figure 2) and ROX (Supplementary Figure 3) detection. The same serum sample-derived cDNA was subsequently analyzed by qPCR for validation, and the results are presented in Figs. 8C. Among the 50 clinical serum samples, qPCR identified 35 positive samples, corresponding to a positivity rate of 70% (35/50). The RT-LAMP-CRISPR/Cas12a assay showed complete agreement with qPCR results, achieving 100% overall concordance (Table 1). Specifically, all 35 qPCR-positive samples were correctly identified by the RT-LAMP-CRISPR/Cas12a assay (true positives = 35), and all 15 qPCR-negative samples were correctly classified (true negatives = 15), with no false-positive or false-negative results observed. The PPA and NPA, together with their 95% CIs, were calculated using the Clopper–Pearson exact method. The RT-LAMP-CRISPR/Cas12a assay showed a PPA of 100% (95% CI: 89.7%–100%) and an NPA of 100% (95% CI: 79.4%–100%) compared with qPCR. The detailed comparison of RT-LAMP-CRISPR/Cas12a and qPCR results, including CT values for individual clinical serum samples, is provided in Supplementary Table 7.

**Table 1:** Comparison of results from RT-LAMP-Cas12a and qPCR methods for detecting clinical serum samples (n=50).

	qPCR positive	qPCR negative	Total
RT-LAMP-Cas12a positive	35	0	35
RT-LAMP-Cas12a negative	0	15	15
Total	35	15	50

Note: Data are presented as number of samples.

Positive percent agreement (PPA) = 100% (95% CI: 89.7%–100%), Negative percent agreement (NPA) = 100% (95% CI: 79.4%–100%), Overall percent agreement (OPA) = 100% (95% CI: 92.9%–100%).

## 4 Discussion

The continuous mutation and recombination of PRRSV pose substantial challenges for disease prevention and control, highlighting the urgent need for rapid, sensitive, and specific diagnostic approaches. In this study, a novel nucleic acid detection platform for PRRSV-2 was developed by integrating RT-LAMP amplification with CRISPR/Cas12a-mediated target recognition. This platform enables rapid amplification and highly specific detection of target nucleic acids under simple operating conditions and allows visual interpretation of results through fluorescence signals.

Currently, conventional PRRSV diagnostic approaches, including virus isolation, antigen detection, and molecular assays such as RT-qPCR, provide high sensitivity and specificity but generally require sophisticated thermal cycling instruments, trained personnel, and relatively long processing times, limiting their application in rapid on-site diagnosis. Serological methods, such as enzyme-linked immunosorbent assay (ELISA), are primarily used for antibody detection and are insufficient for the early diagnosis of acute infection (Biernacka et al., 2016). Isothermal amplification technologies, including LAMP and RPA, reduce equipment requirements and enable decentralized testing; however, their application alone may be compromised by nonspecific amplification and false-positive results caused by primer-dependent recognition. In this study, the CRISPR/Cas12a system was incorporated to enhance detection specificity. The crRNA directs Cas12a to recognize the target sequence within LAMP amplicons, thereby activating its trans-cleavage activity and cleaving single-stranded DNA (ssDNA) reporter molecules to generate detectable fluorescent signals (Nguyen et al., 2020). The combination of LAMP amplification and crRNA-mediated Cas12a recognition provides a dual-targeting mechanism that improves analytical specificity and reduces the risk of nonspecific detection (Pang et al., 2020). Recently, integrated platforms combining isothermal amplification with CRISPR-based detection have been successfully applied for the diagnosis of various swine pathogens, including swine influenza virus (SIV) (Guo et al., 2026), Seneca virus A (SVA) (Jiang et al., 2025), African swine fever virus (ASFV) (Yang et al., 2022), porcine circovirus type 2 (PCV2) (Lei et al., 2022), and PEDV (Liu et al., 2022), further demonstrating the broad potential of this strategy for veterinary pathogen detection.

Regarding analytical performance, the detection limit of the developed assay was 9.17 × 10⁻¹ copies/μL, which was lower than that reported for the RPA-CRISPR/Cas13a method (172 copies/μL) developed by Chang et al. (2020) and the multi-crRNA RPA-CRISPR assay (6 copies/μL) reported by Guo et al. (2026), and was comparable to the single-copy detection capability reported by Liu et al. (2021). The enhanced sensitivity of the present assay may be attributed to the multi-primer recognition strategy of LAMP, in which four to six primers simultaneously target multiple regions of the target sequence, thereby improving amplification efficiency and reducing nonspecific amplification. In addition, Bst DNA polymerase used in LAMP is generally more cost-effective than the recombinase-based enzyme mixture required for RPA, providing advantages for large-scale implementation in resource-limited settings. Nevertheless, RPA exhibits a shorter reaction time (approximately 30 min versus 50 min for LAMP) and enables simplified one-step workflows, which may reduce the risk of aerosol contamination. Therefore, LAMP and RPA each possess distinct advantages depending on the application scenario. In this study, LAMP was selected due to its high sequence recognition capability, cost-effectiveness, and potential advantages for detecting low-abundance nucleic acid targets.

Regarding methodological limitations, several aspects should be considered. First, the sensitivity evaluation in this study was performed using plasmid DNA (pMD19T-M) as the template. Compared with single-stranded RNA, double-stranded circular plasmid DNA exhibits greater structural stability, and previous studies have suggested that LAMP amplification may show higher sensitivity toward cDNA templates than RNA templates. Therefore, the LoD determined using plasmid DNA may not fully reflect the detection performance when applied to clinical RNA samples and could potentially underestimate the actual detection limit in clinical settings. Second, the current specificity evaluation was limited to four porcine viruses, including HP-PRRSV, PEDV, PRV, and PPV. Further validation against additional common co-infecting pathogens, such as PCV2, SIV, different PPV subtypes, and PRRSV-1, is required to comprehensively assess the specificity and applicability of this assay. Third, the present assay is designed for the specific detection of PRRSV-2. As a single-target detection system, it cannot simultaneously differentiate PRRSV-2 from other pathogens in mixed infections. There is a possibility of missed detection due to the presence of variant strains with mutations in the crRNA binding sites. Therefore, future development of multiplex detection platforms may further expand its clinical application potential. Furthermore, direct comparisons of LoD values among different studies should be interpreted with caution. Variations in target materials (plasmid DNA, *in vitro*-transcribed RNA, or viral isolates), LoD determination criteria (95% detection probability versus complete positivity among tested replicates), and the number of validation replicates can substantially influence the reported sensitivity values. Therefore, LoD comparisons should be considered as relative indicators of analytical sensitivity and order-of-magnitude differences rather than strict numerical benchmarks.

Regarding clinical applicability, several limitations should be acknowledged. The clinical validation in this study was performed using only 50 serum samples collected from a single farm, with a positivity rate of 70%. Therefore, the observed 100% agreement with qPCR (PPA = 100%, NPA = 100%) may not be directly generalizable to populations with different epidemiological characteristics, including variations in PRRSV prevalence, viral genotypes, and herd management practices. In addition, the current evaluation was limited to serum samples, whereas PRRSV can also be detected in other biological specimens, such as oral fluids, nasopharyngeal swabs, semen, and feces. The composition and complexity of these sample matrices vary substantially; for example, oral fluids may contain nucleases, fecal samples contain abundant polysaccharides and humic substances, and semen contains high concentrations of proteins and lipids. These components may interfere with nucleic acid amplification and influence assay performance. Moreover, differences in viral distribution and viral load among sample types may further affect detection sensitivity. Therefore, additional independent validation using diverse clinical matrices is required before broader application of this assay. Although the developed assay reduces instrument requirements and enables rapid signal visualization, it still requires an upstream RNA extraction step (approximately 20 min), involving either a benchtop centrifuge or a commercial extraction kit. Thus, it does not yet achieve a fully integrated point-of-care testing (POCT) workflow with a true “sample-in, result-out” format. Furthermore, the open-tube amplification procedure may introduce a potential risk of aerosol contamination. Accordingly, the current assay is more appropriately positioned as a rapid molecular diagnostic tool for primary veterinary laboratories or mobile testing platforms rather than a completely laboratory-independent POCT solution. Future improvements integrating simplified extraction procedures, closed-tube detection formats, and portable readout devices may further enhance its field applicability.

Despite these limitations, the developed assay provides several advantages for field-oriented PRRSV detection. The detection results can be visualized using portable UV lamps, blue-light devices, or even natural light when combined with ROX probes, without requiring sophisticated laboratory equipment. These features make the assay particularly suitable for rapid screening applications in resource-limited primary veterinary laboratories and large-scale pig farms. Future studies should focus on several aspects to further improve the robustness and applicability of this platform: (1) conducting large-scale clinical validation using samples from different geographical regions, age groups, and infection stages; (2) performing comprehensive cross-reactivity evaluations covering major PRRSV-1 and PRRSV-2 lineages; (3) systematically assessing analytical performance across diverse clinical sample matrices; (4) developing simplified nucleic acid extraction or extraction-free sample preparation strategies; (5) establishing closed-tube detection formats to minimize the risk of aerosol contamination; and (6) integrating multiple CRISPR effector systems (e.g., Cas12a- and Cas13a-based detection) or multiple specific crRNAs to enable multiplex pathogen detection.

## 5 Conclusion

This study established a CRISPR/Cas12a-based detection platform integrated with RT-LAMP amplification for rapid PRRSV diagnosis. The developed assay demonstrated high analytical sensitivity and showed no detectable cross-reactivity with the tested swine viruses, including PEDV G2b, PRV HNJY, and PPV SD-1. Further validation using broader PRRSV genetic variants, PRRSV-1 strains, and additional co-circulating pathogens is required to fully characterize its diagnostic performance. The fluorescence-based detection strategies enabled sensitive, rapid, and visual identification of PRRSV-2, overcoming some limitations associated with conventional diagnostic methods, including dependence on specialized equipment and prolonged processing times. This platform provides a potential technical approach for improving rapid PRRSV detection and supporting on-site surveillance and control in swine production systems.

## Statement for studies in humans/animals

Not applicable. This research involved neither human subjects nor animal experiments.

The serum samples were collected from pigs on a commercial farm as part of routine management and disease surveillance, with no experimental procedures performed on live animals specifically for this research. Therefore, no ethical approval for human or animal studies was required.

## Funding

This work was supported by the 10.13039/501100012166National Key Research and Development Program of China (2021YFD1301200), Henan Province Major Industrial Key Technology Research Open Competition Project (261000110600), the program for Cultivating Young Backbone Teachers in Higher Education Institutions in Henan Province (2024GGJS152), the Key Project of International Science and Technology Cooperation in Henan Province (251111520200), Henan Provincial Science and Technology Research Project (222102110232).

## CRediT authorship contribution statement

**Congcong Li:** Writing – original draft, Project administration, Funding acquisition, Data curation, Conceptualization. **Changsheng Sun:** Writing – review & editing, Resources, Data curation. **Qiuliang Xu:** Writing – review & editing, Funding acquisition, Data curation.

## Declaration of competing interest

The authors declare that they have no conflict interests to declare.

## Appendix Supplementary materials

Image, application 1
